# Voriconazole pseudotoxicity: a case report highlighting the importance of sampling timing in TDM interpretation by clinical pharmacists

**DOI:** 10.3389/fmed.2026.1878131

**Published:** 2026-07-22

**Authors:** Xiao-Ming Deng, Yuan Xu, Zhi-Qi Xie, Jun-Jie Xu, Kai-Li Mao, Hua-Yu Sun

**Affiliations:** 1 Department of Pharmacy, The First People's Hospital of Wuyi County, Jinhua, China; 2 Department of Infectious Diseases, The First People's Hospital of Wuyi County, Jinhua, China; 3 Department of Medical School, Hangzhou City University, Hangzhou, China; 4 Department of Pharmacy, The Quzhou Affiliated Hospital of Wenzhou Medical University, Quzhou People's Hospital, Quzhou, China

**Keywords:** clinical pharmacist, dose optimization, sampling time, therapeutic drug monitoring, voriconazole

## Abstract

This case report describes a 57-year-old male patient who sought medical attention due to severe pneumonia and respiratory failure. The initial empirical antibiotic treatment was ineffective. Bronchoscopy and lung alveolar lavage fluid culture confirmed a case of *Aspergillus fumigatus* infection. Antifungal treatment with the conventional dose of VRZ was administered, but the patient continued to have a fever, and the trough concentration of VRZ detected exceeded the upper limit of the normal range. The clinical pharmacist discovered that the blood sample collected for TDM of this patient was taken 2 h after medication administration rather than the trough concentration. Based on the data of Aspergillus resistance monitoring network and the phenomenon that the patient’s blood drug concentration did not match the clinical efficacy, the clinical pharmacist recommended increasing the dose of VRZ and suggested re-testing the trough concentration of VRZ after the blood drug concentration reached a steady state. The patient rapidly achieved a fever reduction and was ultimately cured. The re-tested blood drug concentration was within the normal range. This case demonstrates that clinical pharmacists play an indispensable role in the quality control of therapeutic drug monitoring. They will standardize the sampling time, interpret the test results in combination with the clinical condition, adjust the dose based on pharmacokinetic principles, strengthen interdisciplinary cooperation, and optimize antifungal treatment.

## 1 Introduction

Invasive pulmonary aspergillosis (IPA) is a life-threatening opportunistic infection, particularly prevalent in immunocompromised hosts and critically ill patients. The mortality rate associated with IPA remains unacceptably high, ranging from 50 to 90%, depending on the patient population and the timeliness of diagnosis (1). For decades, voriconazole (VRZ) has been the cornerstone of antifungal therapy for IPA, demonstrating superior efficacy and improved survival compared to conventional amphotericin B (2). Despite its clinical utility, VRZ presents significant therapeutic challenges due to its complex pharmacokinetic (PK) profile. VRZ exhibits nonlinear PKs due to saturation of its metabolic pathways, primarily mediated by cytochrome P450 (CYP) isoenzymes, specifically CYP2C19 (the major pathway), CYP3A4, and CYP2C9 (3). This results in highly unpredictable interand intra-individual variability in plasma concentrations. Genetic polymorphisms in CYP2C19 significantly influence drug metabolism; “poor metabolizers” are at risk for supratherapeutic levels and toxicity, while “rapid/ultrarapid metabolizers” may experience subtherapeutic levels and treatment failure (4, 5).

To navigate this narrow therapeutic window, Therapeutic Drug Monitoring (TDM) is recommended in selected clinical scenarios, particularly for patients at high risk of suboptimal exposure or toxicity, as reflected in guidelines from the Infectious Diseases Society of America (IDSA) and the Chinese Pharmacological Society (1, 6). The recommended therapeutic trough concentration (Cmin) of VRZ is generally between 1.0–5.5 mg/L (or 0.5–5.0 mg/L depending on the guideline), as achieving this range is associated with improved clinical response and reduced hepatotoxicity and neurotoxicity (6, 7). However, the success of TDM is highly dependent on pre-analytical variables, particularly the timing of blood sampling. Measuring a non-trough level provides limited value for dose adjustment, as concentrations during the absorption or distribution phase do not reflect steady-state drug exposure. A common clinical error is drawing blood during the peak phase or at a random time, which falsely elevates the reported value and may lead to inappropriate dose reduction or premature discontinuation of therapy (8).

We present a compelling case of a patient with severe IPA who exhibited a paradoxical clinical scenario: a reported “toxic” VRZ level but worsening fever. A clinical pharmacist’s intervention to audit the sampling time not only saved the patient from unnecessary dose reduction but also identified the need for a higher dose. This case serves as a model for the value of clinical pharmacy services in optimizing antimicrobial stewardship and patient safety.

## 2 Case description

### 2.1 Patient presentation and history

A 57-year-old Asian male (height 170 cm, weight 67.5 kg) was admitted to the respiratory intensive care unit (ICU) with a 1-day history of severe cough, chest tightness, and shortness of breath. His medical history was significant for type 2 diabetes mellitus diagnosed 4 years prior, for which he had been non-adherent to insulin therapy (self-discontinued).

### 2.2 Initial clinical findings and diagnosis

**Figure 1:**
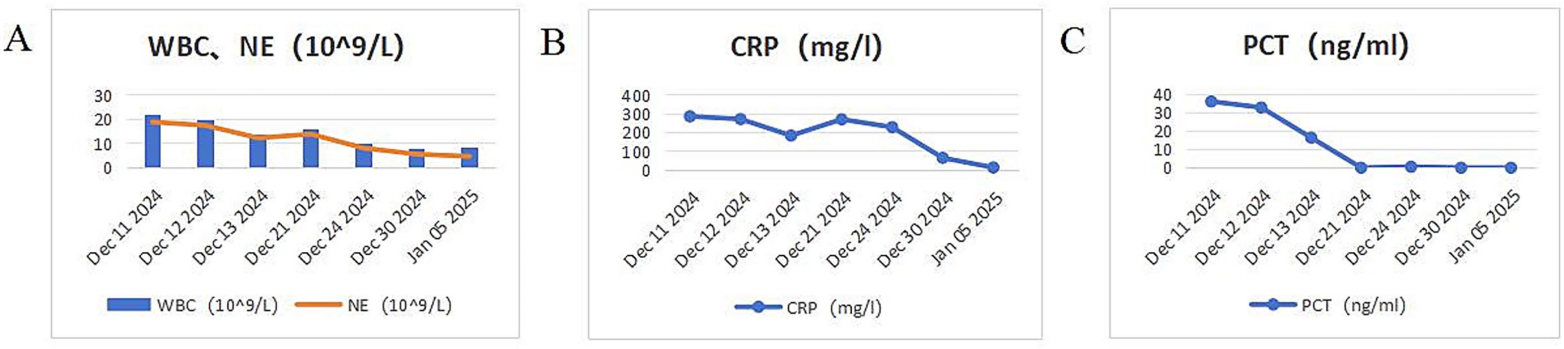
Changes in relevant inflammatory markers during the patient’s treatment, including **(A)** white blood cell and neutrophil counts from routine blood tests, **(B)** high-sensitivity C-reactive protein levels, and **(C)** procalcitonin levels.

**Figure 2:**
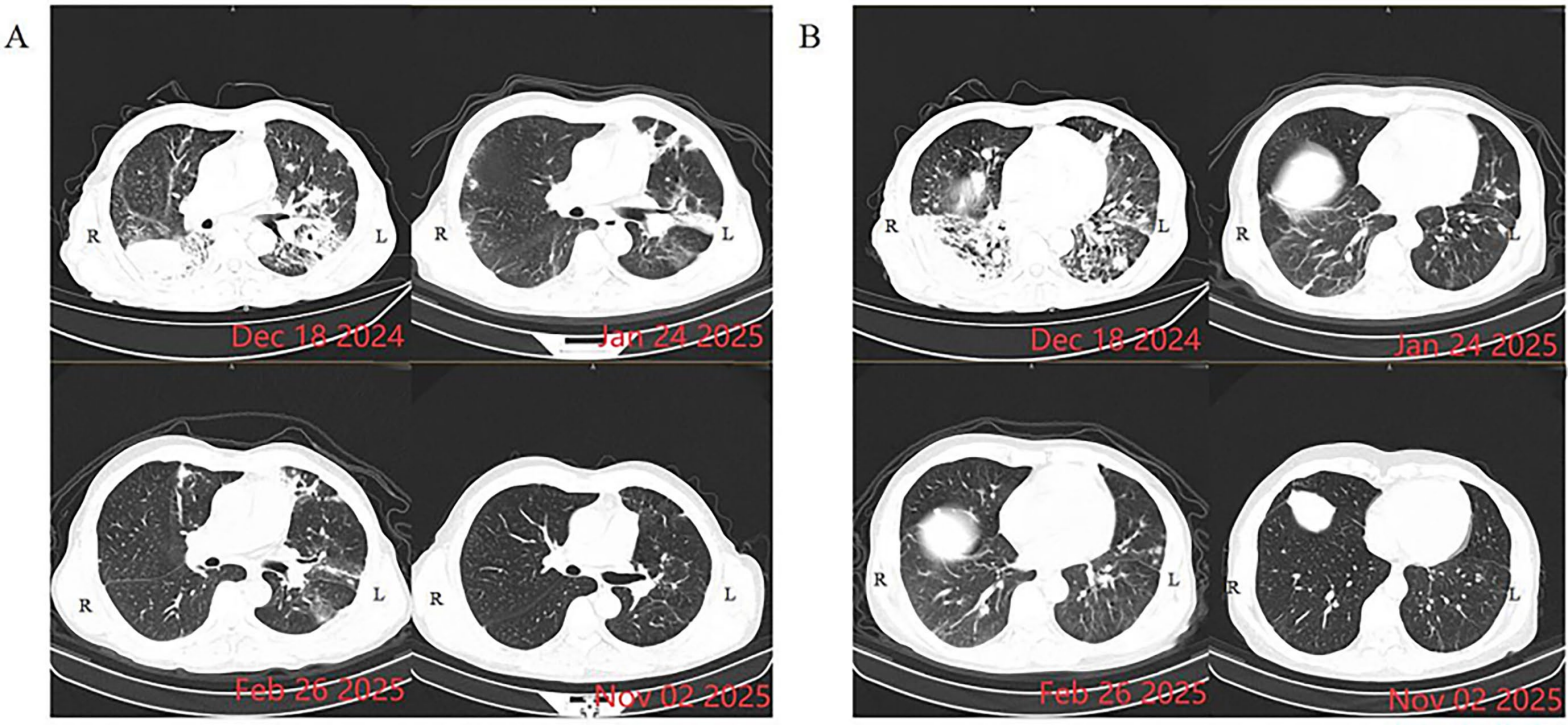
Changes in CT images of the left **(A)** and right **(B)** lungs of the patient with invasive pulmonary aspergillosis before and after treatment. On December 18, 2024, the imaging report indicated multiple patchy opacities in both lungs, with small cavity formation in some lesions presenting as air-crescent signs, more prominent in the right lung, suggestive of Aspergillus infection. On January 24, 2025, chest CT showed significant improvement of the infection in the right lung, but progression of the lesion in the left upper lobe, presenting as a large area of high-density opacity. A follow-up CT on February 26 showed improvement of the left upper lobe lesion. On November 2, follow-up imaging showed significant improvement in both lungs.

Upon admission, the patient was critically ill. Vital signs revealed tachycardia (119 bpm), tachypnea (33 breaths/min), and hypoxia (oxygen saturation 89% on room air). Blood pressure was 126/80 mmHg, and temperature was 37.1 °C (afebrile on admission but febrile later). Inflammatory markers: WBC 22.0 × 10^9/L, CRP 287.2 mg/L, Procalcitonin (PCT) 35.99 ng/mL (Figure 1). Chest CT revealed bilateral multifocal infiltrates suggestive of infection (Figure 2). The admission Diagnosis included severe pneumonia and respiratory failure.

### 2.3 Initial management

The patient was started on high-flow nasal cannula (HFNC) oxygen (30 L/min, 60% FiO2). Resuscitation included aggressive IV fluids, insulin drip for DKA, potassium repletion, and sodium bicarbonate for acidosis. Empiric broad-spectrum antibiotics (Piperacillin-Tazobactam 4.5 g q8h plus Doxycycline 100 mg/q12h) were initiated (Table 1).

**Table 1:** The usage, dosage and pharmacological effects of the drugs.

Drugs	Dosage	Frequency	Route of medication	Pharmacological action	Approval number	Manufacturers	Medication time
Piperacillin-Tazobactam	4.5 g	q8h	Intravenous drip	Antibacterial drugs	H20073604	Ruiyang Pharmaceutical Co., Ltd.	December 11, 2024–December 16, 2024
Doxycycline	100 mg	q12h	Oral	Antibacterial drugs	H20030627	Dequan Pharmaceuticals (Jiangsu) Co., Ltd.	December 11, 2024–December 16, 2024
Meropenem	1 g	q8h	Intravenous drip	Antibacterial drugs	H20163392	Shandong Luo Xin Pharmaceutical Group Co., Ltd.	December 16, 2024–December 26, 2024
Voriconazole	400 mg	q12h	Oral	Antifungal drug	H20243933	Wuhan QiRui Pharmaceutical Co., Ltd.	December 18, 2024–December 19, 2024
Voriconazole	200 mg	q12h	Oral	Antifungal drug	H20243933	Wuhan QiRui Pharmaceutical Co., Ltd.	December 19, 2024–December 26, 2024
Voriconazole	300 mg	q12h	Oral	Antifungal drug	H20243933	Wuhan QiRui Pharmaceutical Co., Ltd.	December 26, 2024–January 18, 2025
Piperacillin-Tazobactam	4.5 g	q8h	Intravenous drip	Antibacterial drugs	H20073604	Ruiyang Pharmaceutical Co., Ltd.	Dec 262,024-Dec 28 2024

### 2.4 Therapeutic course and diagnostic hurdles

Despite initial resuscitation, the patient remained febrile with persistent hypoxia. Extensive workup for alternative pathogens was negative: COVID-19 PCR (Day 2), respiratory pathogen panel (27 types, Day 2). A repeat Chest CT (Day 7) showed progression of bilateral infiltrates with a halo sign suggestive of angioinvasive aspergillosis.

On Day 8, a bronchoscopy with bronchoalveolar lavage (BAL) was performed. The BAL fluid PCR returned positive for *Aspergillus fumigatus* and *Klebsiella pneumoniae*. Culture confirmed *A. fumigatus*.

On Day 9, the patient was started on Voriconazole (Vfend) tablets: Loading dose 400 mg q12h x 2 doses, followed by maintenance of 200 mg q12h. The antibacterial regimen was escalated to Meropenem (1 g q8h).

### 2.5 The clinical dilemma

The patient continued to spike fevers. A serum voriconazole level was drawn on Day 13 (at 11:00 a.m.) and resulted as 6.4 mg/L. The target normal range in the hospital lab was 0.5–5.0 mg/L. The treating physician was concerned that the patient was experiencing supratherapeutic levels (potential toxicity) despite the lack of clinical response, and considered reducing the dose.

### 2.6 Pharmacist intervention

The clinical pharmacist reviewed the case. Key observations were:

Clinical-Efficacy Mismatch: The patient was not improving clinically (persistent fever, CRP [229 mg/L on Day 13]).

Toxicity Absent: The patient had no signs of hepatotoxicity (LFTs normal) or neurotoxicity (no visual disturbances or encephalopathy).

The Critical Finding: The pharmacist inquired about the exact time of blood sampling. The nurse confirmed the blood was drawn at 11:00 a.m., approximately 10–11 h after the previous evening dose but only 2 h after the morning dose (given at 9:00 a.m.). This was a peak/trough hybrid, not a true trough.

### 2.7 Recommendation and action

The pharmacist advised the team: Do not reduce the dose. The 6.4 mg/L level is likely a peak level and falsely elevated. Re-educate staff: Strict protocol for TDM must be followed: “Draw trough level just before the next dose (i.e., 30 min prior to the morning dose).” Should re-sampling be necessary, voriconazole trough concentrations should be reassessed after steady-state is attained (4–7 days). In cases where the steady-state voriconazole trough concentration is below the lower boundary of the target normal range, or in the presence of an insufficient clinical response, a 50% upward adjustment of the voriconazole maintenance dose is recommended.

### 2.8 Outcome

Day 15 (Follow-up): The patient defervesced (temperature normalized) within 48 h of the dose increase. Day 17 (Verification): Repeat trough level on 300 mg q12h was 3.2 mg/L (steady-state). Discharge (Day 25): The patient showed adequate response to therapy for infection control, afebrile, normal oxygen saturation, and CRP dropped to 65.2 mg/L. He was discharged on oral voriconazole 300 mg q12h. Follow-up (Day 77): Repeat Chest CT showed significant resolution of pulmonary infiltrates. During the follow-up period, the patient expressed great satisfaction with our treatment.

## 3 Discussion

This case provides a powerful illustration of the “value add” of clinical pharmacists in the critical care setting. While TDM is a mature science, its real-world execution is often flawed. We highlight three key areas where pharmacist intervention directly impacted patient outcomes: (1) Quality control of the TDM process, (2) Reconciliation of PK/PD principles with clinical observation, and (3) Guideline-recommended dose optimization.

### 3.1 The high cost of pre-analytical errors in TDM

TDM is only as good as the sample quality. Studies show that up to 71.5% of TDM samples in busy clinical wards are drawn incorrectly (9). The current case exemplifies the classic error of “random” sampling. The voriconazole concentration-time profile follows a distinct pattern: peaks at 1–2 h post-oral dose and declines gradually (10). A sample drawn at 11 a.m., while post-peak, remained well above trough levels. Acting solely on the 6.4 mg/L result could have led the physician to reduce or stop the drug, risking fungal progression and death. The pharmacist’s role as a “diagnostician of the process” was essential, supporting literature that pharmacist-led TDM services enhance sampling accuracy and interpretation (11).

### 3.2 Clinical vs. laboratory discordance: a pharmacist’s Lens

A key skill in clinical pharmacy is to distinguish between drug toxicity and disease progression. The patient continues to have a fever and elevated inflammatory markers, which are typical symptoms of active infection rather than voriconazole toxicity (such as encephalopathy or visual hallucinations) (12). The corrected trough value of 3.2 mg/L at 300 mg q12h falls within the treatment window of the Chinese guidelines (0.5–5.0 mg/L).

### 3.3 Dose optimization strategy: the 50% increment rule

Our intervention—increasing the dose by 50% (from 200 mg to 300 mg)—is supported by clinical pharmacology principles. Because voriconazole exhibits non-linear (Michaelis–Menten) kinetics, increasing the dose results in a more than proportional increase in serum concentration. The guideline by Chen et al. (6) specifically recommends: “If the clinical response is poor or the trough concentration is below the target, the maintenance dose can be increased by 50%. We successfully applied this rule. The resulting level remained within the safe range (3.2 mg/L) and yielded clinical cure.

### 3.4 Comparison with existing literature

Our case complements recent publications on the complexity of VRZ TDM. For instance, López-Hernández et al. (13) recently reported a case of unexpected VRZ toxicity due to interaction with Nirmatrelvir/Ritonavir, where TDM was essential to identify a paradoxical increase in levels. That case, like ours, underscores that TDM interpretation requires a deep understanding of drug–drug interactions and patient-specific variables. Furthermore, while a 2023 randomized controlled trial by Veringa et al. suggested that TDM-guided dosing did not significantly improve composite outcomes compared to standard dosing in hematological patients (14), our case argues for a different nuance: TDM is necessary, but it must be “intelligent TDM.” The Veringa trial relied on standard TDM; however, if sampling errors are present in the “real world” (as they often are), the data becomes noise. Our case shows that a pharmacist-led audit transforms raw data into actionable wisdom.

### 3.5 Implications for clinical practice

Standardization of Sampling: Hospitals must implement strict protocols specifying that VRZ trough levels must be drawn within 30 min before the morning dose. Nurses require periodic training. Interprofessional Rounding: This case demonstrates why pharmacists must be part of the ICU rounding team. The delay in recognizing the sampling error would have been prolonged without direct pharmacist access to the chart and the nursing staff. Clinical response (or lack thereof) and laboratory values should be viewed as complementary pieces of information. A mismatch between them should trigger a pharmacist-led review of the TDM process. Clinical pharmacists play an increasingly vital role in chronic disease management, including infection management and respiratory disease care (15–17). Our case reinforces this evidence by demonstrating how pharmacist-led TDM interpretation and medication optimization can prevent inappropriate therapeutic decisions and improve patient outcomes.

### 3.6 Limitations

As a single case report, the findings are not generalizable to all populations. The patient was a known drinker (potential CYP450 inducer), which may have contributed to the relatively low levels on standard dosing. We did not perform CYP2C19 genotyping, which could have definitively explained the metabolic phenotype (though the clinical response to 300 mg suggests he was a normal or rapid metabolizer). Additionally, the cost-effectiveness of daily TDM vs. standard care was not assessed. While *Aspergillus fumigatus* was consistently recovered from both BAL and sputum specimens, our center does not offer MIC, GM, or G-testing, thereby limiting our capacity to formally exclude acquired resistance. However, given the patient’s marked improvement following therapeutic dose augmentation, clinically significant resistance appears improbable.

### 3.7 Future research perspectives

Future studies should focus on the development of Bayesian dosing software integrated with electronic health records. Such software could prompt clinicians on correct sampling times and predict individualized doses based on a single level. Moreover, the role of the pharmacist as a “TDM steward” should be formally evaluated in prospective trials measuring the reduction in sampling errors and improvement in clinical outcomes.

## 4 Conclusion

This case report demonstrates that therapeutic failure of voriconazole is often not a failure of the drug, but a failure of drug monitoring. A clinical pharmacist successfully averted a potential medical error by identifying an inappropriately timed TDM sample that falsely suggested toxicity. By reconciling the laboratory data with the patient’s deteriorating clinical status, the pharmacist re-sampled a true trough, identified subtherapeutic exposure, and implemented a guideline-recommended 50% dose escalation, leading to complete clinical resolution of invasive pulmonary aspergillosis.

We strongly advocate for the integration of clinical pharmacists into ICU multidisciplinary teams to lead TDM services, provide real-time interpretation, and ensure the safety and efficacy of high-risk medications like voriconazole.

## Data Availability

The original contributions presented in the study are included in the article/supplementary material, further inquiries can be directed to the corresponding authors.
